# Advances in antioxidant activities of edible mushroom polysaccharides: status, influencing factors, mechanisms, and applications

**DOI:** 10.3389/fnut.2026.1806859

**Published:** 2026-05-29

**Authors:** Yao Zhu, Ping He, Ruihua Zhao, Yueqin Liu, Xiaolong He, Kai Wang, Xiang He, Kenan Wang, Yuting Zhu, Xiaopeng Gao, Pengfei Jin

**Affiliations:** Shaanxi Key Laboratory of Research and Utilization of Resource Plants on the Loess Plateau, Engineering Research Center of Microbial Resources Development and Green Recycling of the University of Shaanxi Province, Research and Development Centre of Ecological and Sustainable Application of Microbial Industry of the Loess Plateau in Shaanxi Province, College of Life Science, College of Yan’an Medical, Yan’an University, Yan’an, Shaanxi, China

**Keywords:** antioxidant, application, edible mushroom, influencing factors, mechanism, polysaccharides

## Abstract

Polysaccharides represent one of the most significant bioactive constituents found in edible mushroom. These compounds exhibit a range of physiological activities, including antioxidant, anti-aging, antibacterial, and anti-inflammatory effects, as well as hypoglycemic and hypolipidemic properties. This article mainly reviews the antioxidant activity of polysaccharides, analyzes the current research status of antioxidant properties of various edible mushroom polysaccharides, and systematically elucidates the influencing factors of antioxidant activity, the antioxidant mechanisms, and their applications in the fields of medicine, pharmaceuticals, food, and cosmetics.

## 1 Introduction

Due to their high protein, dietary fiber, essential amino acids, vitamins, and minerals, and their low fat and calorie content, edible mushroom have steadily gained attention in health-related endeavors as health consciousness has grown (1–4). Polysaccharides, triterpenoids, various amino acids, and proteins are among the active components found in edible mushroom (5–8). Among those, polysaccharides have a wide range of applications and are crucial in the food, cosmetics, pharmaceutical, tobacco, and agricultural sectors (9, 10). Numerous bioactivities, including immunomodulation, anticancer effects, antibacterial activity, antioxidant properties, hypolipidemic effects, and hypoglycemic effects, have been reported for edible mushroom polysaccharides (11–15).

The human body continually generates free radicals due to a variety of conditions. Oxidative stress, carried on by an excess of free radicals, can lead to cellular aging, inflammation, cancer, and other chronic illnesses (16). By interacting with free radicals, removing excess reactive oxygen species (ROS) from the body, and raising intracellular antioxidant enzyme levels to support physiological balance, polysaccharides have anti-oxidant effects (17, 18). Malondialdehyde (MDA) levels dramatically increase in diabetic nephropathy, and enzymes like superoxide dismutase (SOD) are not released enough to eliminate oxygen-free radicals. As a result, oxygen-free radicals cause renal harm to tubular epithelial cells and change tubular structure and function through peroxidation reactions with their membrane structures. *Ganoderma lucidum* polysaccharide peptides (GL-PP) have been shown by Fang et al. to significantly increase the activities of superoxide dismutase and glutathione peroxidase in renal tissue of rats with proteinuric nephropathy, while markedly reducing urinary levels of thiobarbituric acid reactive substances, a classic biomarker of oxidative stress. These findings suggest that GL-PP and GL-PP_2_ exert renoprotective effects by effectively alleviating renal oxidative stress injury induced by proteinuria (19). The intracellular polysaccharides (IPS) from *Pleurotus eryngii* SI-04 and their purified fractions IPS-1 and IPS-2 showed good *in vitro* antioxidant properties, according to Zhang et al. They could chelate Fe^2+^ and scavenge hydroxyl radicals, superoxide anions, hydrogen peroxide, and 1,1-diphenyl-2-picryl-hydrazyl radical (DPPH) radicals. Additionally, these polysaccharides exerted hepatoprotective effects by ameliorating the liver index, serum biochemical parameters, and hepatic oxidative stress status, as well as alleviating histopathological damage in mice with CCl₄-induced liver injury (20). Synthetic free radicals like DPPH and 2, 2′-Aazino-bis (3-ethylbenzothiazoline-6-sulfonic acid) (ABTS•^+^) react with polysaccharide scavengers. They provide benefits such as high sensitivity, stability, convenience of use, and quick evaluation of *in vitro* radical scavenging capacity. However, because they are unable to accurately replicate the intracellular oxidative milieu, they have limited physiological importance (21). Hydroxyl radicals, on the other hand, are actual reactive oxygen species that exist *in vivo* and are directly connected to oxidative damage, including DNA damage and lipid peroxidation, making them more physiologically significant (22). Combining these three techniques improves the biological significance of findings, gives a more thorough representation of a sample’s *in vitro* antioxidant potential, and provides a theoretical framework for further cellular and animal research.

Based on where they originate, food antioxidants can be divided into two categories: natural antioxidants and chemically manufactured antioxidants. Major chemically synthesized antioxidants include butylated hydroxytoluene, butylated hydroxyanisole, propyl gallate (PG), tert-Butylhydroquinone and others. Tea polyphenols, vitamin E, rosemary, licorice antioxidants, bamboo leaf antioxidants, and phytic acid are the principal natural antioxidants (23). Hepatotoxicity, nephrotoxicity, and contact dermatitis are only a few of the serious health hazards associated with chemically manufactured antioxidants. Even though natural antioxidants are generally safer, consuming too much of them can still have negative effects or even be toxic. Poly-saccharides from edible mushrooms are harmless, non-irritating, and devoid of harmful side effects. They moisturize the skin and provide anti-aging and antioxidant benefits. They have anti-inflammatory and immune-modulating qualities in addition to bolstering the body’s antioxidant defenses. Polysaccharides from edible mushrooms have shown a great deal of promise for both research implementation and practical application. In order to provide theoretical underpinnings and references for the effective use of these active components, this review highlights recent research on the antioxidant efficacy of active chemicals in edible mushroom polysaccharides.

## 2 The state of antioxidant research on polysaccharides found in common edible mushrooms

### 2.1 Polysaccharides from *G. lucidum*

The edible and medicinal fungus *G. lucidum* has a long history of usage in medicine. It is widely used in the prevention and treatment of many ailments and is listed in the Chinese Pharmacopoeia as a traditional Chinese medicinal material. It has benefits like no harmful side effects and no drug resistance in humans (24–26).

Li et al. (27) prepared oxidized *G. lucidum* polysaccharide-based hydrogels and investigated their antioxidant activity and efficacy in diabetic wound repair *G. lucidum*. These hydrogels could efficiently scavenge •OH and •O₂^−^ radicals, reduce intracellular ROS levels, enhance antioxidant capacity by activating related signaling pathways, and alleviate oxidative stress injury, thereby providing a safe and efficient novel strategy for the antioxidant repair of chronic diabetic wounds.

Li et al. (28) used 5 × FAD mice (carrying five familial Alzheimer’s disease mutations) as an animal model to investigate the antioxidant and neuroprotective effects of *G. lucidum* polysaccharides (GLPS). GLPS significantly increased SOD levels, decreased MDA content, and alleviated oxidative stress *G. lucidum*.

Zhang et al. (29) found that oxygen free radicals are critical factors contributing to multiple organ dysfunction syndrome (MODS). After administration of *G. lucidum* polysaccharides (GLP) to MODS model mice, the results demonstrated that SOD and catalase (CAT) can convert the highly toxic superoxide anion radicals into O₂ and H₂O₂, thereby detoxifying peroxide ions and attenuating free radical–induced damage, which may provide a theoretical basis for the application of GLP in the treatment of MODS *G. lucidum* (29).

### 2.2 Polysaccharides from *Agaricus bisporus*

Because of its high protein content, low fat level, and abundance of vitamins such as niacin and riboflavin, as well as polysaccharides including *β*-glucan, heteropolysaccharides, and proteoglycans, *A. bisporus*, a fungal species of the genus *Agaricus* within the family *Agaricaceae*, is quite popular. Additionally, it is among the most extensively grown types (30).

Liu et al. (31) extracted polysaccharides from *A. bisporus* collected in Xizang and found that they exhibited excellent antioxidant activity. These polysaccharides could effectively increase the levels of SOD and GSH while decreasing MDA content in liver tissue, providing a feasible option for the treatment of drug-induced liver injury (31).

According to the findings of Jelisić et al. (32) two stressors—Nosema ceranae infection and cypermethrin exposure—both trigger intestinal oxidative and energetic stress in honey-bees, which in turn impairs colony viability and elevates individual mortality rates. *A. bisporus* polysaccharide extracts show strong antioxidant activity that preserves ecological variety and safeguards honeybees health by efficiently scavenging DPPH and hydroxyl radicals.

According to Jiang et al. (33), X-ray radiation in mice impairs bone marrow function to produce immunological and hematopoietic suppression as well as testicular oxidative stress, apoptosis, and other damages. *A. bisporus* polysaccharides enhance blood cell counts, lower MDA, raise antioxidant enzymes like SOD, preserve testosterone levels, and lessen radiation-induced testicular damage. Mice are significantly protected against X-ray-induced damage by their antioxidant-related actions.

### 2.3 Polysaccharides from *Tremella fuciformis*

*Tremella fuciformis* is considered a superior yin-nourishing agent and is prized as a valuable culinary ingredient and medicinal herb. *Tremella* polysaccharides (TPs) are used as ingredients in meals, drinks, cosmetics, and medical supplies.

Ge et al. (34) prepared the high-molecular-weight *T. fuciformis* polysaccharide (TFP) by fermentation using *T. fuciformis* strain XY, and investigated it *in vitro* antioxidant activity. The results showed that TFP could effectively scavenge superoxide anion radicals and hydroxyl radicals in a concentration-dependent manner. The maximum scavenging rates against superoxide anions and hydroxyl radicals reached 69.34 and 71.00%, respectively, indicating favorable *in vitro* antioxidant activity (34).

Ma et al. (35) extracted TPs using an ultrasonic-assisted technique and examined how they inhibited the browning of fresh-cut apples and potatoes. The findings demonstrated that these TPs improved hardness and soluble solids content to improve refrigerated taste quality while lowering browning severity and polyphenol oxidase activity in fruits and vegetables. Their film-forming qualities produced oxygen barrier effects, preventing browning, while their antioxidant qualities inhibited oxidase activity and decreased quinone products.

Xu et al. (36) demonstrated that TFP could reduce colonic myeloperoxidase activity, serum diamine oxidase activity and D-lactate levels in colitis mice, alleviate intestinal oxidative damage and inflammatory infiltration, as well as attenuate DSS-induced colonic oxidative stress and inflammatory responses.

### 2.4 Polysaccharides from *Pholiota nameko*

A valuable edible and therapeutic fungus are the *P. nameko*. It has a unique phenotype during growth, which is a slick secretion on the surface of the cap that is sticky to the touch and has a smooth, reviving feeling when eaten.

To examine the antioxidant and wound-healing qualities of *P. nameko* polysaccharides (PNPs), Sung et al. (37) synthesized PNP using ethanol precipitation at 40, 60, and 80% concentrations. Using 3-(4,5-Dimethylthiazol-2-yl)-2,5-diphenyltetrazolium bromide assays (MTT) and *in vitro* scratch tests, they evaluated the effects on L929 fibroblast proliferation and migration, as well as lowering capacity and hydroxyl radical scavenging ability.

Li et al. (38) prepared PNPs using the hot water extraction and ethanol precipitation method. Assay results showed that this polysaccharide could enhance the scavenging ability of fermented soybean milk against DPPH radicals, superoxide anion radicals and hydroxyl radicals.

Su et al. (39) extracted PNPs by the water extraction and ethanol precipitation method, and crude polysaccharides were then obtained by freeze-drying. The PNP demonstrated a DPPH radical scavenging rate of 72.7% at 2 mg/mL and a hydroxyl radical scavenging rate of 53% at 4 mg/mL, according to assays. The polysaccharide at the same dose showed greater cell survival rates in the repair group compared to the control group using an H_2_O_2_-induced oxidative damage model in L929 cells, further demonstrating its antioxidant potential.

### 2.5 Additional polysaccharides

Yang et al. (40) found that polysaccharides from *Lactarius hatsudake* exhibited strong *in vitro* antioxidant activity. The polysaccharides showed stronger scavenging ability against DPPH and ABTS radicals than hydroxyl radicals, and presented a similar tendency to vitamin C at a polysaccharide concentration of 5 mg/mL.

Sun et al. (41) isolated and purified four polysaccharide conjugates from the fruiting bodies of *Auricularia polytricha* using water extraction and ethanol precipitation combined with column chromatography, and evaluated them *in vitro* antioxidant activities. The results showed that all four polysaccharides scavenged hydroxyl radicals, superoxide anion radicals, and chelated Fe^2+^ in a concentration-dependent manner, and the higher the uronic acid content, the stronger the antioxidant activity.

El-Maradny et al. (42) investigated the crude extracts and polysaccharides from *A. bisporus* and *Pleurotus ostreatus*, and evaluated their antioxidant activities using DPPH and ABTS assays. The results showed that *A. bisporus* polysaccharides exhibited the strongest DPPH radical scavenging activity, with a scavenging rate of 93.73% and an half maximal effective concentration value of 0.19 mg/mL. *P. ostreatus* polysaccharides also showed excellent radical scavenging effects, and the antioxidant activities of both polysaccharide extracts were significantly higher than those of the crude extracts. In summary, many edible mushroom polysaccharides exhibit favorable antioxidant activity and play vital roles in effectively regulating cellular functions and maintaining organismal health. Li et al. (43) found that the *A. bisporus* polysaccharide fraction with stronger antioxidant activity exerted better protective effects on the liver and kidney of D-galactose-induced mice, as well as superior regulatory effects on dyslipidemia and stronger hypolipidemic activity. According to Qu et al. (44) *Laetiporus sulphureus* polysaccharides disrupted the redox balance in tumor cells by inhibiting SOD activity, depleting GSH, and increasing MDA levels, thereby inducing excessive oxidative stress and ultimately leading to tumor cell apoptosis. *Hericium erinaceus* polysaccharides attenuated oxidative stress and cytokine production in the colonic tissue of DSS-induced colitis mice, alleviated inflammatory damage to the intestinal mucosa, and improved intestinal microbial dysbiosis (45). Consequently, as a fundamental biological activity, the antioxidant effect of polysaccharides does not act independently, but interacts and synergizes with other physiological functions to jointly maintain cellular and organismal homeostasis.

## 3 Influencing factors of antioxidant activity of edible mushroom polysaccharides

### 3.1 Types of edible mushrooms

The antioxidant activity of edible mushroom polysaccharides is closely related to the species of edible mushroom. With pure polysaccharides from its fruiting bodies showing 98.09% ABTS^+^ scavenging efficiency and 75.88% DPPH scavenging efficiency, *G. lucidum* polysaccharides showed high antioxidant activity (46). *Russula vinosa* polysaccharides exhibit ABTS^+^ scavenging activity approximately 38.8 times higher than that of *Auricularia auricula* (47). *T. fuciformis* polysaccharides have strong moisturizing effects but a weaker antioxidant capacity, and *A. bisporus* polysaccharides are slightly weaker than *G. lucidum* but stronger than *P. ostreatus* (42, 48).

### 3.2 Extraction and preparation methods of polysaccharides

The conditions under which polysaccharides are extracted and prepared have a significant impact on the antioxidant activity of edible mushroom polysaccharides. Water extraction and ethanol precipitation are conventional methods for polysaccharide extraction. As research interest in edible mushrooms has increased in recent years, various extraction techniques have been developed. Li et al. (43) used hydrochloric acid to extract polysaccharides from *A. bisporus* and found that these polysaccharides also exhibited strong antioxidant activity. Zhang et al. (49) employed six methods to extract polysaccharides from *P. nameko* residues: alkali extraction, enzymatic extraction, ultrasonic-assisted extraction, hot water extraction, microwave-assisted extraction, and acid extraction. A comparative analysis indicated that hot water-alkali-assisted extraction yielded polysaccharides with outstanding antioxidant activity, due to their high xylose content and low molecular weight. In addition to extraction methods, drying methods also considerably affect the structure and antioxidant activity of polysaccharides. According to Fan et al. (50), *Schizophyllum commune* polysaccharides prepared by freeze-drying showed the highest contents of total sugar and uronic acid, exhibited a loose and porous structure under electron microscopy, and possessed stronger antioxidant activity than those obtained by other drying methods. Water extraction and ethanol precipitation are commonly used for polysaccharide extraction. This method causes less damage to polysaccharides and can retain their native structure to the greatest extent. However, ethanol concentration also significantly influences the antioxidant activity of polysaccharides. Kang et al. (51) used gradient ethanol precipitation (40, 50, 60, 70, and 80%) to extract *A. bisporus* polysaccharides, and found that those obtained with 70% ethanol exhibited the highest uronic acid content, relatively low molecular weight, and the most significant overall antioxidant activity.

In conclusion, the antioxidant activity of polysaccharides is jointly regulated by multiple factors. In addition to the inherent differences in antioxidant capacity among edible mushrooms, changes in extraction and preparation methods essentially alter the physicochemical properties and structures of polysaccharides. Molecular weight, monosaccharide composition, polysaccharide content, three-dimensional structure, microstructure, and other characteristics all significantly affect the antioxidant activity of edible mushroom polysaccharides.

## 4 Edible mushroom polysaccharides’ antioxidant mechanism

Numerous signaling pathways depend on ROS, which are produced during regular cellular metabolism. ROS build up excessively as a result of cellular stress and decreased enzyme activity, upsetting the dynamic balance between oxidation and antioxidation. This causes oxidative stress in cells, which results in necrosis and tissue damage. This can lead to a number of disorders, including cancer, inflammation, high blood pressure, hyperglycemia, and other chronic conditions. A class of chemicals known as antioxidants takes part in, reduces, and gets rid of reactive compounds produced by oxidative stress. Eating naturally occurring antioxidants can efficiently control the antioxidant system and lessen the harm that reactive oxygen species do to cells and the body. Studies show that polysaccharides from edible mushroom have strong antioxidant properties as natural antioxidants (52). Their two main mechanisms of action are “direct radical scavenging” and “indirect activation of the body’s antioxidant enzyme system”, with the two having complementary benefits. The following are the particular mechanisms:

First of all, it stops the oxidative stress chain reaction by immediately removing excess free radicals from the body. According to pertinent research, the primary agents causing oxidative damage are free radicals, which include hydroxyl radicals, superoxide anion radicals, ABTS cation radicals, and DPPH radicals. By binding with these free radicals and going through electron transfer reactions, edible mushroom polysaccharides can directly eliminate them and lessen the attacks of free radicals on cells. Relevant studies indicate that *Cordyceps militaris* polysaccharides exhibit excellent scavenging effects on OH^−^ radicals, O_2_^−^ radicals, ABTS^+^ radicals, and DPPH radicals (53). Chou et al. (54) conducted *in vitro* antioxidant tests using *P. nameko* polysaccharides precipitated with 40, 60, and 80% ethanol solutions of varying purities. Results indicated that PNP80 exhibited the strongest DPPH and ABTS^+^ scavenging activities, as well as the highest Fe^2+^chelation capacity, demonstrating the most potent antioxidant properties. Chen et al. (55) investigated the effects of ultrasonic-assisted extraction on *Flammulina filiformis* polysaccharides. Comparing this method with traditional hot water extraction, they found ultrasonic-assisted extraction exhibited stronger reducing power and scavenging activity against DPPH radicals, hydroxyl radicals, and superoxide anion radicals. Research indicates that the antioxidant potency of mushroom polysaccharides is closely linked to their structural characteristics and determined by multiple factors, rather than being controlled by a single variable. They discovered that ultrasonic-assisted extraction demonstrated greater reducing power and scavenging activity against DPPH radicals, hydroxyl radicals, and superoxide anion radicals when compared to conventional hot water extraction.

Second, polysaccharides can improve the body’s antioxidant defense capacities by indirectly activating the antioxidant enzyme system. Strong antioxidant enzyme systems, including SOD, CAT, peroxidase (POD), glutathione peroxidase (GSH-PX), and others, are naturally present in organisms. In order to preserve the internal oxidative-antioxidative dynamic equilibrium and guarantee regular biological processes, these antioxidant enzymes cooperate and work in concert. The last byproduct of lipid peroxidation, MDA, is a sign of oxidative damage to cells. Fruiting body polysaccharides have been shown by Cai et al. (56) to scavenge HO, DPPH, and O^2−^ radicals, lower MDA levels, increase SOD, CAT, and GSH-PX activity, and shield zebrafish embryos from oxidative damage.

**Figure 1:**
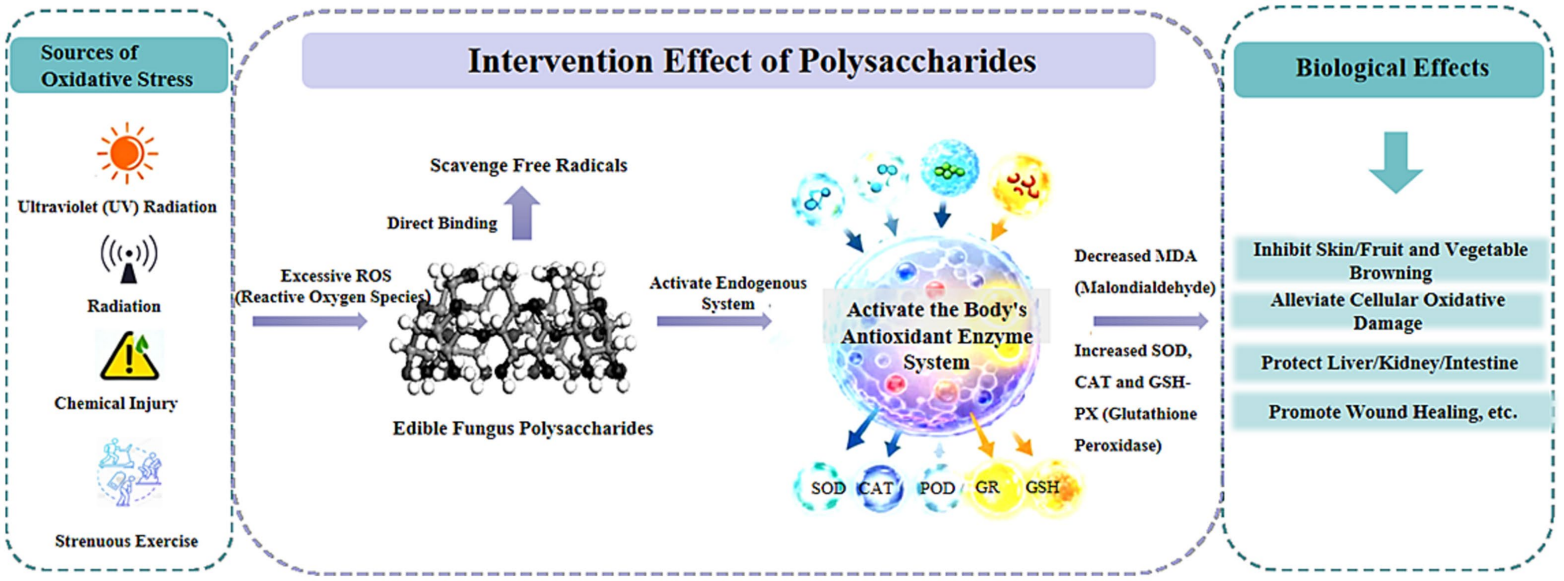
Schematic diagram of edible mushroom polysaccharides’ antioxidant mechanism.

Xu et al. (57) degraded *G. lucidum* polysaccharides using ultrasonic treatment. In a mouse model of high-fat diet, the activities of SOD and GSH-Px as well as MDA content in serum and liver were determined. The results showed that degraded *G. lucidum* polysaccharides significantly increased the activities of antioxidant enzymes and reduced lipid peroxidation levels. Its antioxidant effect was markedly stronger than that of undegraded polysaccharides, and it also ameliorated lipid metabolism disorders in liver tissue. Ma et al. (35) looked at how *T. fuciformis* polysaccharides prevented fresh-cut fruits and vegetables from browning. In comparison to the control group, fresh-cut apples and potatoes treated with *T. fuciformis* polysaccharides at a mass concentration of 4.0 mg/mL showed polyphenol oxidase (PPO) activities that were 0.44 and 0.66 times higher, respectively. Whereas the degree of browning was 0.27 and 0.64 times more than that of the control group, respectively. Consequently, TPs show a significant ability to prevent fresh-cut fruit and vegetables from browning. Their antioxidant qualities, which obstruct the oxidation reactions mediated by PPO, are primarily responsible for this function, which indirectly shields plant cells from oxidative damage (Figure 1).

## 5 Uses for polysaccharide antioxidation in edible mushrooms

### 5.1 Pharmaceutical and medical uses

In order to cure wounds in experimental mice, Abdulhameed and Kadhim created an ointment combining petroleum jelly and *P. ostreatus* extract (58). The *P. ostreatus* extract was found to have antioxidant, antimicrobial, and anti-inflammatory properties as well as the capacity to promote angiogenesis and collagen synthesis, suggesting the possibility of wound healing and repair. Hou used fermented products from *C. militaris*, *G. lucidum*, and *P. eryngii* to create a public health beverage (59). MTT techniques and DPPH radical scavenging tests showed antioxidant and anticancer properties in all three. The restorative benefits of freeze-dried powder from these three fermented mushroom products on CCl₄-induced acute liver injury in mice were confirmed by acute liver injury studies. Han et al. (60) added *C. militaris* poly-saccharides to probiotic yogurt in 2020. Serum/liver biochemical markers, liver indices, and the mice’s body weight were all measured. Hematoxylin–eosin staining was used to look for pathological alterations in liver tissue that had been processed into paraffin slices. The findings demonstrated that probiotic yogurt added with variable concentrations of *C. militaris* polysaccharides improved mice’s liver damage caused by alcohol to differing degrees. Fungal polysaccharides are widely used in the prevention and treatment of agricultural diseases, which goes beyond research on humans and animals. Zang et al. (61) investigated the regulatory effects of *Lentinus edodes* polysaccharides on antioxidant capacity in wheat by means of seed dressing. The results showed that seed dressing with *L. edodes* polysaccharides significantly increased the activities of phenylalanine ammonia-lyase, peroxidase and superoxide dismutase in wheat leaves, effectively reduced malondialdehyde content, alleviated membrane lipid peroxidation damage, and enhanced the antioxidant capacity of plants. This effect was most significant at 7 days after seedling emergence.

### 5.2 Uses in food

Edible mushrooms are popular because they are naturally high in protein and dietary fiber and low in calories and fat (62). Their antioxidant qualities can be used as food additives to increase nutritional value and prolong shelf life in bread, pastries, and other items (63, 64). Wang et al. (65) added polysaccharides from *A. polytricha* to set-style yogurt to improve its texture and flavor, while enhancing the body’s antioxidant and anti-damage effects via the Keap1/Nrf2/HO-1 signaling pathway. Di Renzo et al. (66) pointed out that mushroom fermented beverages are produced through microbial fermentation using fruiting bodies, mycelia or extracts of edible and medicinal fungi. They are rich in polyphenols, polysaccharides and other bioactive substances, and exhibit significant antioxidant activity, which can effectively improve the antioxidant function of beverages. A series of products have been developed, including mushroom wine, mushroom beer, mushroom kombucha and non-alcoholic fermented beverages, which possess both nutritional and health-promoting values. Bai created a powder that replaces whole grain meals with polysaccharides from *Tricholoma matsutake* (67). Created a nutrient-rich, satisfying, health-promoting, fat-reducing meal replacement powder with immune-boosting effects by refining the recipe and evaluating its immunological activity. Chen et al. (68) extracted a novel bioactive *T. fuciformis* gum (TFG) from the fruiting bodies of *T. fuciformis*. The polysaccharide content in TFG reached as high as 73.9%, and it showed excellent water solubility, colloidal properties and stability. The sweets have anti-inflammatory, gut microbiota-improving, and antioxidant qualities. More importantly, TFG maintained stable antioxidant activity during all stages of *in vitro* digestion, suggesting that it can be used as a potential food thickener and antioxidant.

### 5.3 Uses in cosmetics

**Figure 2:**
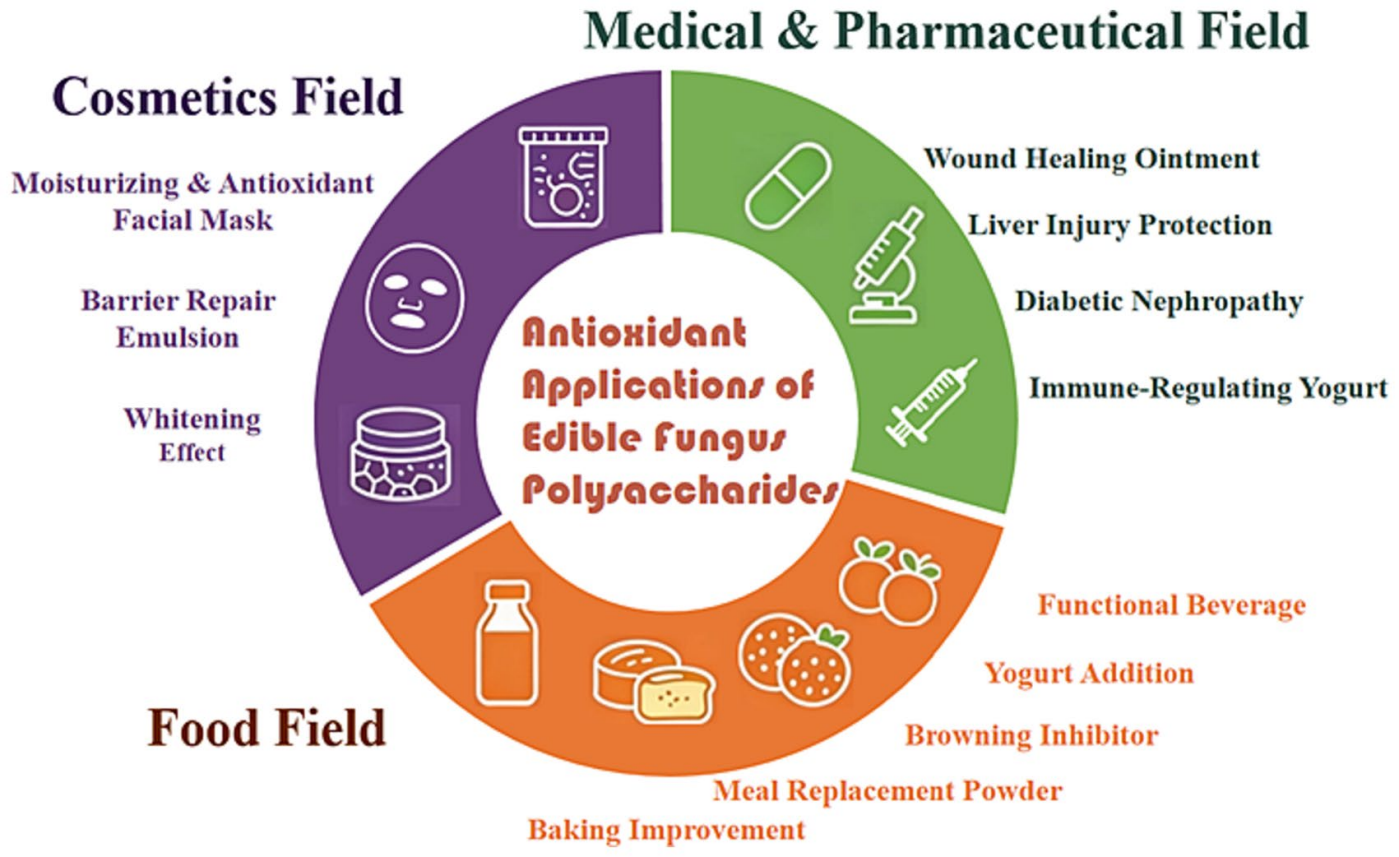
Various uses of edible mushroom polysaccharides in antioxidant studies.

With its many advantages for skin health, including antioxidant protection, anti-aging properties, moisturization, and brightness, edible mushroom are a perfect cosmetic component (69). Oxidative stress and tissue damage are caused by factors like Ultraviolet (UV) exposure, environmental stresses, and sleep deprivation that enhance the formation of free radicals. Dryness, sagging, pigmentation, and accelerated skin aging are symptoms of this disruption of normal cellular growth, which results in cell lysis and death (70). Wang et al. (71) used the polysaccharides from *L. edodes* to create a multipurpose mask gel with antioxidant and moisturizing properties. A 0.1% *T. fuciformis* polysaccharide emulsion was used by Cao et al. (72) to strengthen the skin barrier, lower the production of inflammatory factors IL-1β, IL-6, IL-10, and TNF-*α*, significantly reduce skin protein glycation, increase skin hydration, improve elasticity and brightness, and significantly inhibit skin redness caused by damage. Tyrosinase and tyrosinase-related protein 1 are two examples of proteins linked to melanin formation that are stimulated by reactive oxygen species, which upset the redox balance of melanocytes and increase melanin synthesis. According to Kim et al. (73), *Grifola frondosa* polysaccharides suppress tyrosinase activity and have skin-beautifying effects by inhibiting important components of the tyrosinase and tyrosine-related protein synthesis pathways, such as cAMP response element-binding protein 1 and microphthalmia-associated transcription factor (Figure 2).

## 6 Discussion and prospects

The current state of antioxidant capacities across diverse edible mushroom was examined in this evaluation of the antioxidant functions of edible mushroom polysaccharides, which revealed varying levels of antioxidant activity among various species. Polysaccharide antioxidant efficacy is correlated with mushroom species, according to *in vitro* free radical scavenging tests; *Pleurotus bisporus* has a higher scavenging ability than other fungi. Additionally, there is a strong correlation between the antioxidant capacity of polysaccharides and the extraction techniques used; polysaccharides extracted with differing amounts of ethanol have different antioxidant capacities. The antioxidant capacity of polysaccharides varies depending on the enzymatic hydrolysis conditions. Polysaccharides’ physicochemical characteristics are inextricably linked to their antioxidant potential; polysaccharides with high uronic acid concentration and low molecular weight exhibit better antioxidant capacities. Another important aspect affecting antioxidant activity is the structure of polysaccharides. The connection between antioxidant action and structure is still not well understood, despite tremendous advancements in polysaccharide antioxidant research. Finding the genes that affect antioxidant activity is still quite difficult. Future research should delve deeper into the antioxidant characteristics of edible mushroom, concentrating on the screening of antioxidant genes and the structure–function link.

By scavenging excess free radicals and stimulating antioxidant enzyme activity, polysaccharides efficiently reduce the generation of reactive oxygen species and minimize cellular stress and physiological damage. Polysaccharides have anti-inflammatory, anti-aging, antibacterial, lipid-lowering, and hypoglycemic benefits in addition to their antioxidant qualities. These processes work together to keep the body stable and in balance. As a result, they are widely used in a variety of industries, including food, cosmetics, pharmaceuticals, and agriculture. Despite being used in many different industries, polysaccharide antioxidants still have a small market share. To encourage wider usage across other domains, more research is required.
